# Gaps in the Evidence on Population Interventions to Reduce Consumption of Sugars: A Review of Reviews

**DOI:** 10.3390/nu10081036

**Published:** 2018-08-08

**Authors:** Sharon I. Kirkpatrick, Amanda Raffoul, Merryn Maynard, Kirsten M. Lee, Jackie Stapleton

**Affiliations:** 1 School of Public Health and Health Systems, University of Waterloo, 200 University Ave. W., Waterloo, ON N2L 3G1, Canada; araffoul@uwaterloo.ca (A.R.); kirsten.lee@uwaterloo.ca (K.M.L.); 2 Meal Exchange, 401 Richmond St. W., Toronto, ON M5V 3A8, Canada; merryn@mealexchange.com; 3 University of Waterloo Library, 200 University Ave. W., Waterloo, ON N2L 3G1, Canada; jstapleton@uwaterloo.ca

**Keywords:** sugars, sugar-sweetened beverages, population interventions, taxation, food environments, nutrition education, review of reviews

## Abstract

There is currently considerable attention directed to identifying promising interventions to reduce consumption of sugars among populations around the world. A review of systematic reviews was conducted to identify gaps in the evidence on such interventions. Medline, EMBASE CINAHL, and the Cochrane Database of Systematic Reviews were searched to identify systematic reviews published in English from January 2005 to May 2017 and considering research on interventions to reduce sugar intake. Twelve systematic reviews that considered price changes, interventions to alter the food available within specific environments, and health promotion and education programs were examined. Each of the identified reviews focused on sugar-sweetened beverages (SSBs). The existing literature provides some promising indications in terms of the potential of interventions to reduce SSB consumption among populations. However, a common thread is the limited scope of available evidence, combined with the heterogeneity of methods and measures used in existing studies, which limits conclusions that can be reached regarding the effectiveness of interventions. Reviewed studies typically had limited follow-up periods, making it difficult to assess the sustainability of effects. Further, there is a lack of studies that address the complex context within which interventions are implemented and evaluated, and little is known about the cost-effectiveness of interventions. Identified gaps speak to the need for a more holistic approach to sources of sugars beyond SSBs, consensus on measures and methods, attention to the implementation of interventions in relation to context, and careful monitoring to identify intended and unintended consequences.

## 1. Introduction

A growing body of research supports associations between intake of sugars and their key sources, including sugar-sweetened beverages (SSBs), with negative health outcomes, including obesity, dental caries, and cardiometabolic syndrome [1,2,3]. In response to this still emerging but growing literature on the deleterious implications of high consumption of sugars, national and international bodies have issued recommendations urging limits to intake. For instance, the World Health Organization recommends reduced intake of free sugars (“monosaccharides and disaccharides added to foods and beverages by the manufacturer, cook or consumer, and sugars naturally present in honey, syrups, fruit juices and fruit juice concentrates”) throughout the life course, with a reduction to less than 10 percent of total energy intake among both adults and children [2]. A conditional guideline recommending that sugar intake be limited to less than five percent of total energy intake was based on ecological studies on sugar intake and dental caries. Similarly, the most recent Dietary Guidelines for Americans defined healthy eating patterns as those that limit added sugars (which are “sugars that are either added during the processing of foods, or are packaged as such, and include sugars (free, mono- and disaccharides), syrups, naturally occurring sugars that are isolated from a whole food and concentrated so that sugars are the primary component (e.g., fruit juice concentrates), and other caloric sweeteners”) to less than 10 percent of calories per day [3]. 

On the basis of increased understanding of the relevance of sugars to health and emerging guidelines and recommendations, there is considerable attention placed on identifying effective interventions to de-incentivize the purchase and consumption of sugars among populations around the world [1,4,5]. However, understanding the implications of such interventions is complex due to an array of contextual factors that have the potential to interact with elements of interventions, possibly leading to unintended consequences, including synergistic effects as well as policy resistance and failure [6]. Further, the literature in this area is relatively nascent but rapidly emerging, posing a challenge to researchers and policymakers alike in terms of translating research into practice [7]. To shed light on the state of the research on the effectiveness of interventions to reduce sugar consumption among populations, a review of systematic reviews of the international literature was conducted to identify gaps in this evidence base, as well as considerations for future research and for policies and programs to reduce sugar intake.

## 2. Materials and Methods

PRISMA guidelines [8] applicable to a narrative review of reviews were followed. The search methods were developed in collaboration with a research librarian (JS). Relevant databases including MEDLINE, EMBASE, and the Cumulative Index to Nursing and Allied Health Literature (CINAHL) were searched, with limits applied to retrieve records for review articles only. The Cochrane Database of Systematic Reviews was also searched. Relevant database subject headings and keywords were identified to capture reviews considering interventions at various levels (e.g., regional, national, and global) aimed at supporting reductions in sugar consumption among populations. Frameworks including NOURISHING [9] and the International Network for Food and Obesity/Non-communicable Diseases Research, Monitoring and Action Support (INFORMAS) [10], as well as relevant documents (e.g., Public Health England’s reports on sugar reduction) [1], were consulted to guide the search terms. A priori areas of interest included interventions affecting prices and availability of sugary foods and drinks and/or alternatives, health promotion and educational programs, product reformulation, and initiatives related to nutrition labeling and health claims, as well as those targeting exposure or resilience to advertising and marketing. The full MEDLINE search strategy as well as the results of the final searches are available in the Supplementary Materials. The search was initially conducted in November 2015 and updated in May 2017.

**Figure 1:**
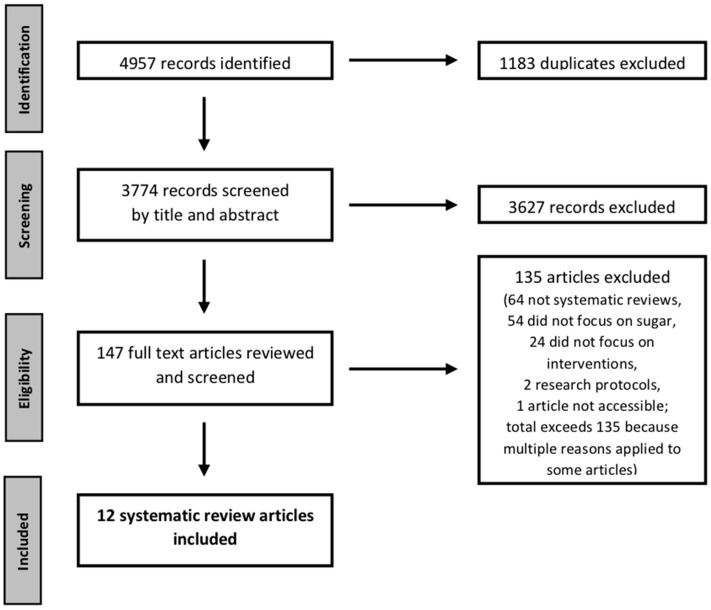
PRISMA diagram illustrating screening process for selection of review articles examining the effectiveness of interventions for reducing sugar consumption.

Figure 1 outlines the screening process. Trained researchers (AR, MM, and KML) conducted screening, with two researchers independently reviewing each record. Criteria for inclusion included: (i) reported use of systematic search methods and a description of the search strategy (e.g., search terms and databases used, and screening process); (ii) a focus on the effectiveness of interventions and strategies to reduce sugar intake (including specific sources of sugars such as SSBs), with reviews that included examination of at least one intervention study related to sugars considered; and (iii) publication in peer-reviewed journals in English from January 2005 on, because the interventions of greatest interest and relevance are likely to have emerged and been tested in the past decade or so. A pilot test of the process for screening titles and abstracts with 50 records indicated agreement on 92% of inclusion and exclusion decisions, and discrepancies were discussed and resolved. Following independent review of all titles and abstracts, the full text articles were reviewed independently by two researchers against the inclusion criteria (Kappa = 82% for the November 2015 search and 81% for the May 2017 search). 

Twelve review articles [7,11,12,13,14,15,16,17,18,19,20,21] meeting the inclusion criteria were identified. Details for each study were extracted by AR, MM and KML, with review by SIK, to identify the scope/focus of the synthesis, inclusion criteria employed, date ranges covered, numbers of studies reviewed, types of interventions and study designs considered, geographic and population coverage, and main findings and cited limitations. Because of our interest in characterizing the state of the literature and gaps in that literature, we did not conduct a quality assessment for the purpose of excluding lower-quality reviews, but we did examine whether the review authors themselves appraised the quality of primary studies, as well as the funding sources for the reviews.

Based on the final pool of reviews, interventions were categorized into three groups based on their focus: price changes (for example, through taxation) [12,13,14,16,19,20], strategies to alter features of the food environment such as the foods or beverages available within a particular setting [7,11,15,16,17,18,21], and health education and promotion initiatives [7,11,15,16,17,18,21]. These categories are somewhat arbitrary; for example, a change to price could be considered to alter the food environment by shifting affordability and thus perceived availability of a food or beverage. However, a means of categorizing the types of interventions was useful for summarizing the evidence and identifying research gaps. 

Based on the data extraction, we briefly summarize evidence on effectiveness, and then synthesize gaps in the evidence and describe considerations related to research to address these gaps as well as emerging recommendations for policies and programs. 

## 3. Results

### 3.1. Characteristics of the Reviews and the Studies Reviewed

Table 1 provides an overview of the reviews included. Nine of the twelve were published within the past five years (from 2013 on), indicating growing interest in this area. Although the inclusion criteria were not limited to reviews considering SSBs, they were a focus of all included reviews.

Several review authors employed some means of quality appraisal of the studies they reviewed [8,11,12,19,21]. For example, in relation to controlled trials, these appraisals encompassed whether studies were randomized, groups were comparable at baseline, blinding was implemented and clearly described, and dropouts described, as well as the nature of outcome measures utilized, including whether diet assessments were broader than the specific target food/dietary component. Some authors who did not employ quality appraisal noted the lack of an appropriate framework and of consensus on criteria given different study designs. As a result, some included comments on the quality of particular studies (e.g., in terms of the diet assessment method employed) or of the body of research as part of their review rather than conducting a priori quality appraisals (Table 1). The appraisals, formal or otherwise, suggest that the evidence on this topic comes from studies with different levels of robustness. 

Three of the 12 reviews (or their authors) were supported at least in part by funds from the beverage industry (including Coca-Cola, the Union of European Beverages Associations, and the Fundacion Mexicana para la Saulud A.C., which receives research donations from Coca-Cola, PepsiCo, and Pena Fiel) [7,15,16]. Sources of funding were not a major focus of the reviews conducted by these authors. However, Levy et al. [16] noted methodological limitations in a study funded by industry and Gibson et al. [15] cited suggestions that industry-funded studies may show smaller effects than other research. Gibson et al. also noted the potential for publication bias, suggesting that it typically works in the opposite direction of any bias associated with industry funding (with studies showing null effects not reaching the peer-reviewed literature), resulting in an overestimation of positive effects [15].

### 3.2. Evidence on the Effectiveness of Interventions to Reduce Consumption of Sugary Beverages

Of the twelve reviews, six offered insights on interventions to alter price, seven synthesized evidence related to food environment interventions, and seven addressed health promotion and education. Table 2, Table 3 and Table 4 provide summaries by category (for studies addressing multiple types of interventions, salient details are included in each relevant table, though it is not necessarily possible to break down the findings by type of intervention in the case of multicomponent approaches). 

The overall evidence is mixed, with some studies showing that interventions lead to significant reductions in purchasing or intake of SSBs. While positive results have also been found for reductions in caloric intake and weight or body mass index, the evidence is more equivocal for these outcomes. Further, long-term effects of interventions were limited in studies with longer follow-up periods. Overall, the existing data suggest that interventions may be most beneficial for populations considered to be “disadvantaged” (e.g., affected by low socioeconomic status) and provide some indication of potential compensatory and substitution behaviors in response to interventions (e.g., taxes on SSBs may lead to increased consumption of other beverages), but further evidence is needed.

### 3.3. Gaps and Limitations in the Existing Evidence on Interventions to Reduce Consumption of Sugary Beverages

A common thread across reviews was the limited evidence available combined with heterogeneity of methods, which hinders conclusions regarding effectiveness of interventions. Although some authors engaged in methods of synthesis such as meta-analyses, formal approaches were often not undertaken due to heterogeneity. For example, in examining studies on the impact of changes in prices of SSBs on weight outcomes, Cabrera Escobar et al. [14] found that it was not possible to perform a meta-analysis. Heterogeneity extended to study design; the populations and settings in which interventions have been evaluated; and definitions, measures, and methods used to capture intake and related variables. Study designs included randomized clinical trials, cross-sectional and longitudinal observational studies, quasi-experimental studies, and modeling studies. Barriers to conducting controlled trials were noted; for example, Gibson et al. [15] stated “in practice it is often difficult to ensure comparability of groups at baseline, compliance in the intervention group, non-contamination of the control group, and adequate monitoring of diet and lifestyle during the trial”. Limitations of cross-sectional studies, such as inability to establish temporality and causality, were also noted and at least one review indicated that data from such studies might overestimate potential effects [20]. Although there were longitudinal studies, these tended to have limited follow-up periods, posing a barrier to examination of the sustainability of changes in sugar consumption or related outcomes. However, Powell et al. [20], in their review of the influence of price changes through taxes and subsidies on demand and body weight outcomes, noted that, in comparison to previous reviews, they found a larger proportion of longitudinal versus cross-sectional and modeling studies, indicating a positive trend toward the use of prospective research designs. 

Much of the existing research has been conducted in the U.S. The extent to which this body of evidence is transferable to other settings is unclear. In terms of population subgroups, there are few data for young children and pregnant women. Some studies focused on school-aged children and adolescents, but the majority did not distinguish among age groups. Further, there was a noted lack of studies examining interventions targeted to children outside of school settings [11].

Another issue noted was small sample sizes, resulting in studies not adequately powered to detect intervention effects that might truly exist [15,17]. Relatedly, the extent to which interventions studied in controlled trials and experiments can be scaled up and applied to larger populations appears to be unclear. In the area of taxes and subsidies in particular, many studies, while potentially informative, were based on predictive modeling rather than real-world applications and were unable to address context nor the potential feasibility of the interventions tested. Such studies typically did not allow for differentiation of effects by factors such as age, sex or gender, and socio-economic status. To some degree, the lack of information on differential impacts appears to be a function of the available data used for modeling purposes. For example, Powell et al. [20] noted the use of household-level or time series data in studies examining the effect of price on demand or purchasing.

A lack of consensus on definitions of sugars and SSBs was also evident. For example, some studies did not differentiate between regular and diet soft drinks, which complicates interpretations of findings and comparability of study results [15]. There is also a lack of consensus on how to quantify intake amounts and frequency [15,17]. In studies measuring intake, there was typically a reliance on data from 24-h recalls, food records/diaries, food frequency questionnaires, and brief instruments (i.e., screeners). As noted in the reviews, these tools have intrinsic limitations that must be borne in mind when interpreting results. In addition to biases related to misreporting, considerations include whether dietary data were available at repeated time points in prospective studies, sensitivity to changes over time and/or to differences among groups, and the scope of dietary factors assessed. In many studies, the lack of comprehensive dietary data was a barrier to assessing substitution effects [15,17]. It was also suggested that the omission of foods (e.g., snacks) commonly consumed with SSBs (for example, using brief diet assessment tools) could affect reporting of beverage consumption [17]. Self-report questionnaires were also used for related outcomes, such as preferences, with potential reporting biases that can lead to spurious results. In studies examining outcomes related to weight, measures included the prevalence of overweight and obesity, body mass index, and body weight, again affecting the comparability of findings across studies. These outcomes were ascertained using objective as well as self-report measures, with the self-reported data bringing possible biases in terms of misreporting of weight and height.

In addition to variation across studies, there is a lack of research that embraces the complicated context within which complex interventions to influence dietary and related behaviors are implemented. Review authors noted the need for evaluation of interventions that are put into place in the “real world” (outside of controlled/experimental settings and designs), as well as for attention to how interventions interact with contextual factors, such as current and past policies. For example, Levy et al. [16] noted that, although some studies consider more than one policy, “they often do not explicitly consider how the effects of a policy may depend on the other policies in effect or how the effects vary by initial BMI, racial/ethnic group, and SES. Furthermore, the effects of policies may depend on exposure to past policies. The effects of a specific policy may also vary over time”. Cost-effectiveness is another area that requires further research. For example, Avery et al. [11] noted that only one of eight studies included in their review attempted to estimate the cost of implementation.

Conversely, studies on interventions with multiple components are not necessarily able to disentangle effects of each. Understanding the effects of individual components of interventions may not be necessary in cases in which the multi-component approach is retained. However, caution may be warranted when stripping off individual components that have previously been tested as part of a comprehensive approach in which there may be synergistic (or antagonistic) effects. 

## 4. Discussion

We examined 12 systematic reviews that considered evidence on interventions for reducing sugar consumption among populations. Despite a broad search strategy, all of the reviews included focused on outcomes related to consumption and purchasing of SSBs, which is not necessarily surprising given the contributions of sugary beverages to added and total sugar consumption. However, populations are exposed to added and free sugars from a range of other sources, and it is possible that SSBs are becoming less important contributors given intense scrutiny and the implementation of policy measures such as taxation. The focus on SSBs may limit the utility of the current evidence to inform interventions to reduce sugar intake from other sources and to improve the quality of the overall diet. In an examination of international recommendations related to dietary carbohydrates, Buyken et al. [22] found a similar focus on SSBs, with justifications for sugar guidelines often based on observed associations between SSB consumption and disease risk. It is possible that newer studies focusing on sources of sugars aside from SSBs have not yet been incorporated into reviews and, thus, these studies would not have been considered in the current review of reviews. However, the dominant focus on SSBs suggests the need for a more holistic approach to help inform approaches to improve diet quality overall. 

The interventions considered in the reviews included approaches to change consumer behavior by altering price (for example, through taxation), modifying food environments in terms of availability of sources of sugars and alternatives, and health promotion and education. The available evidence provides some indication that these types of interventions have the potential to reduce intake of SSBs and that the effects may be most pronounced for those at greatest risk (e.g., affected by socioeconomic disadvantage or overweight/obesity). Findings are less consistent in terms of effects on caloric intake and body weight. Appraisals employed by review authors suggest that this literature is made up of studies of varying quality, due to methodological limitations and variation across studies. A range of study designs was used, usually with limited follow-up periods and varied definitions, measures, and methods for assessing sugar intake and other key variables. The high degree of heterogeneity led to suggestions that interpretations about the totality of the available evidence be approached with caution. As noted by Althuis et al. [7], “we found considerable variability among primary research studies in terms of designs, definitions of SSB, and definitions of outcomes, which ultimately rendered this research difficult to interpret collectively”. Further, the bulk of the research has been conducted in the USA and coverage of diverse settings and populations is limited. There is also a lack of research that has considered how interventions interact with contextual factors that may influence effectiveness. Other than some examination of potential substitution and regressive effects, there appear to be scarce data on unintended consequences of policies, including at the level of consumers and the food industry. Notably, in an examination of the implications of an excise tax on SSBs implemented in Mexico on purchasing of beverages, Colchero et al. [23] cited unpublished monitoring that reveals aggressive in-store promotion to retain market shares for SSBs.

Based on the gaps identified, several suggestions for improving the evidence on the effectiveness of interventions for reducing intake of sugars emerge, in addition to the need for a focus beyond SSBs. Research examining a broader range of populations and environments (e.g., interventions targeted to children outside of schools), as well as settings outside of the USA is needed. Further, when possible, studies should be designed to allow differentiation of subpopulations (i.e., low-versus medium- to high-income populations) to identify potential differential and regressive effects (e.g., decreased SSB consumption among medium- and high-income subgroups but not low-income groups). Adequate statistical power is needed to test the effects of interventions and to enable accounting for potential mediating variables and confounders. Studies with longer follow-up periods and rigorous measurement of intervention implementation and impact at multiple time points are also needed. Using the least-biased and most reliable measures and methods possible for measuring outcomes, including dietary intake and body weight, should be a goal for any study, and in intervention studies, considering responsiveness of measures to change is also salient. Attention to the process of implementation and the role of contextual factors, such as other policies that may be reinforcing or antagonistic as well as population characteristics that may influence implementation, uptake, and impact, may help policymakers to understand the possible effects of a given intervention or package of interventions in a specific setting. Data on cost-effectiveness of interventions, as well as the potential for scaling up, are also needed to better inform policy decisions.

Overall, the results of this review suggest there remains substantial uncertainty in this literature, but emerging considerations for planning, implementing, and monitoring interventions include the need to appraise the evidence through a lens that considers context, taking into account factors that may affect implementation and effectiveness, as well as the potential for different impacts in relation to sociodemographic and other characteristics. It seems worthwhile for policymakers to consider a package of interventions that are mutually reinforcing, including approaches that target environments (including a variety of settings) and individuals, and that are complementary to existing interventions and other contextual factors. Considering the feasibility of target behaviors, with a staged/modular approach that focuses on one behavior change at a time as well as taking into account current consumption patterns are also warranted. Finally, ongoing evaluation of intended and unintended effects, including substitution and differential effects that could impact health equity, is critical given current gaps in knowledge.

Several considerations should be borne in mind in interpreting this review. We limited our scope to systematic reviews and did not consult primary research articles; thus, our synthesis was limited to the authors’ interpretation of and review of the primary data they included. In some cases, the reviews addressed topics beyond interventions (such as associations between consumption of sugars and weight status) and the number of studies reviewed that specifically addressed interventions could be quite small. It was sometimes challenging to abstract details of findings of these intervention studies in particular. Some of the reviews categorized studies into different types of interventions (as did we), with the potential for masking the impact of multicomponent approaches. Further, as previously noted, the reviews focused specifically on SSBs and thus did not yield insights into interventions to address other key sources of added or free sugars (e.g., baked goods or dairy desserts). We did not exclude reviews in which the authors did not formally assess the quality of the articles they reviewed because such reviews were useful for our main objective of identifying limitations and gaps in the literature. We also did not exclude reviews supported at least in part by industry. Very few of the systematic reviews themselves included consideration of the funding sources for the primary articles that were drawn upon and any potential implications of funding source on study findings and interpretation. Finally, although most of the reviews included were published quite recently, reviews can become outdated quickly given the rapid emergence of new research [7], indicating a need to interpret insights in the context of newly-published evidence. Further, meta-analyses of this literature need to be considered carefully given the observed heterogeneity across studies [24]. 

## 5. Conclusions

Diet and food purchasing behavior are complex phenomena [25,26], and population health interventions are also complicated [26,27]. Thus, it is no surprise that assessing interventions to curb intake of sugars is challenging. Examining the influence of a given intervention on more distal outcomes such as overweight and obesity is also difficult given that body weight is influenced by an extremely complex array of factors [25]. 

Research to address current limitations in the evidence on interventions to reduce intake of sugars can help build a stronger and more cohesive body of literature with which to inform policies and programs. In the interim, the existing studies suggest some promising approaches to promote environments and behaviors consistent with lower SSB intake, although comprehensive monitoring is critical to assess intended and unintended outcomes over the long-term among diverse subgroups of the population. 

## Figures and Tables

**Table 1:** Characteristics of systematic review articles (*n* = 12) including at least one intervention study intended to reduce consumption of sugary beverages.

Authors and Year of Publication	Type/Focus of Synthesis	Inclusion Criteria for Studies Included in the Systematic Review ^1^	Date Range Covered	Relevant (Total) Studies ^2^	Interventions Considered	Study Designs Considered	Geographic/Population Coverage	Quality Appraisal	Funding Source(s) for Review/Authors
Althuis MD, Weed DL. (2013) [7]	Synthesis of SSB ^3^ consumption and relationship with health outcomes, including evidence mapping	Cohort or intervention study Examined relationship between SSB intake/dietary patterns and health outcomes Longitudinal analysis Analysis of SSB consumption	1 January 1966–October 2012	10 (77)	Health education/promotion, food environment	Longitudinal, RCTs ^4^	North America, South America, Europe; Children and adults, aged 6–79	Not stated	Coca-Cola Company
Avery A, Bostock L, McCullough F. (2015) [11]	Qualitative synthesis of interventions to reduce SSB consumption among school-aged children, and associations with body weight	RCTs involving ≥100 healthy weight, overweight and obese children between 2–18 years Focus on lowering intake of sugary beverages Data from control groups provided Change in intake of SSBs and change in body weight reported Intervention ≥6 months long Written in English Lowering intake of SSBs a main outcome	2000–August 2013	8 (8)	Health education/promotion, food environment	RCTs	Brazil, England, Germany, Netherlands, U.S.; Children, aged 2–18 years (mean age 10)	Jadad scale for randomized controlled trials, with scores ranging from 1 (low quality) to 5 (high quality); scores ranged from 2 to 5 (mean score 3.5)	British Dietetic Association
Backholer K, Sarink D, Beauchamp A, Keating C, Loh V, Ball K, Martin J, Peeters A. (2016) [12]	Synthesis of studies that examined the effect of SSB price increases on beverage consumption, purchasing patterns, or body weight outcomes, with focus on differential impacts by socio-economic position	Reported effect of SSB price changes on beverage intake or purchases, energy intake, and/or body weight outcomes in relation to a marker of socio-economic position Conducted in high-income country	Database inception–June 2015	11 (11)	Price changes and taxes	Cross-sectional, modeling	Australia, Ireland, New Zealand, U.K., U.S.; Children and adults (ages not stated)	Quality assessed using a checklist derived from recent reviews of pricing studies, one point per criteria (e.g., evaluation of an actual versus hypothetical tax, long-run input data), with scores ranging from 1 (low) to 7 (high); sensitivity analysis used on studies scoring ≥4 points, 6 of 11 studies received scores of ≥4 points	Australian Research Council, Australian National Preventive Health Agency, Victorian Government Operational Infrastructure Support Program
Bes-Rastrollo M, Sayon-Orea C, Ruiz-Canela M, Martinez-Gonzalez MA. (2016) [13]	Synthesis of studies that examined the association between SSB prices and weight outcomes	Conducted with humans and examining the effect of SSB consumption on weight gain or obesity Written in English, Spanish, or French	Database inception–August 2015	24 (71)	Price changes and taxes	Comparative risk assessment, cross-sectional, longitudinal, micro-simulation models	Australia, India, Ireland, New York City, South Africa, U.K., U.S.; children and adults	Not stated	Spanish Government-Instituto de Salud Carlos III, European Regional Development Fund (FEDER), Navarra Regional Government, University of Navarra
Cabrera Escobar MA, Verrman JL, Tollman SM, Bertram MY, Hofman KJ. (2013) [14]	Meta-analysis of studies examining impact of taxes or price increases on SSB ^3^ intake and body weight	Written in English Primary findings on effect of SSB price changes on intake of SSBs and other drinks, or weight loss, obesity, or BMI	January 2000–January 2013	9 (9)	Price changes and taxes	Cross-sectional, longitudinal	Brazil, France, Mexico, U.S.; Children and adults, all ages	No quality assessment tool was used, but potential for bias in own-price elasticities incorporated into the meta-analysis was considered	International Development Research Centre, Canada
Gibson S. (2008) [15]	Synthesis of the association between SSBs and body weight	Primary studies and reviews Written in English Examined intake of sugary beverages (all cold drinks with added sugars, including soda pop [not diet] and fruit drinks with less than 100% fruit juice) and relationship with body weight, BMI or adiposity in adults or children Cross-sectional, prospective, interventions and RCTs	1994–July 2008	3 (44)	Health education/promotion, food environment	RCTs	Brazil, Canada, Denmark, Germany, Ireland, Netherlands, Norway, Spain, U.K., U.S.; Children, aged 9–18 years	No quality assessment tool was used, but strengths and weaknesses of studies were discussed	Union of European Beverages Associations
Levy DT, Friend KB, Wang YC. (2011) [16]	Examination of effects of policies to reduce SSB consumption among children in schools on weight (no meta-analysis due to heterogeneity of evidence)	Studies examining school nutrition policies and other interventions involving SSBs (carbonated beverages, sports or vitamin beverages, and juice beverages) Written in English	1988–2008	26 (26)	Health education/promotion, food environment, price changes and taxes	Cross-sectional, longitudinal, RCTs	Brazil, Belgium, Canada, Holland, UK, U.S.; Children, grades 1–12	Authors note a focus on the better studies	Robert Wood Johnson Foundation, Fundacion Mexicana para la Salud A.C. (receives research donations from Coca-Cola, PepsiCo, and Pena Fiel)
Malik VS, Schulze MB, Hu FB. (2006) [17]	Synthesis of the association between SSBs and weight gain/obesity (meta-analysis attempted, but heterogeneity prohibitive)	Cross-sectional, prospective cohort, and experimental studies of the intake of SSBs (soft drinks, soda, fruitades, fruit drinks, sports drinks, sweetened iced tea, squashes, and lemonade) and weight gain and/or obesity Written in English Endpoints evaluating body size or weight measurement For prospective cohort studies, a duration of at least 6 months	1966–May 2005	2 (30)	Health education/promotion, food environment	RCTs	Canada, Norway, Spain, U.K., U.S., (many not listed); Children, aged 7–18 years	Methods of dietary assessment used in each study were examined	National Institutes of Health, American Heart Association
Malik VS, Pan A, Willett WC, Hu FB. (2013) [18]	A synthesis of relationship between SSBs and weight gain in adults and children, meta-analysis provided summary estimates and qualitative summary conducted for studies that could not be included in the meta-analysis	Primary research Prospective cohort studies or clinical trials with children or adults Reported any multivariable-adjusted coefficient(s) for the association between SSBs and body weight from prospective cohort studies or any metric for the difference in changes in body weight between intervention and control groups from clinical trials Did not combine SSBs with other beverages, foods, or lifestyle factors as an exposure Had a control group and intervened for at least 2 weeks in clinical trial Written in English	1947–March 2013	5 (32)	Health education/promotion, food environment	RCTs	Brazil, Canada, Denmark, Germany, Netherlands, Switzerland, UK, U.S.; Children, aged 8–16 years	Cochrane Collaboration risk of bias tool (7 domains); risk of bias low or unclear; limited evidence of publication bias	National Institutes of Health
Mazarello Paes V, Hesketh K, O’Malley C, Moore H, Summerbell C, Girffin S, van Sluijs EMF, Ong KK, Lakshman R. (2015) [21]	Synthesis of determinants of SSB consumption among children	Intervention studies (RCTs and non-RCTs) targeting SSB consumption Non-intervention/observational studies examining the association between correlates/determinants and SSB consumption in obese/non-obese children Measured SSB consumption using dietary measures Children <7 years	Until June 2014 (no start date specified)	12 (44)	Health education/promotion, food environment	Cluster RCTs, non-randomized trials, quasi-experimental	Belgium, Spain, Thailand, UK, U.S.; Children, aged 2.3–7 years	Quality assessed using eight items focused on internal validity of studies (e.g., method of randomization, measurement of outcome of interest, retention); approximately half rated as “high” quality	National Institute of Health Research, School for Public Health Research, Centre for Diet and Activity Research, Medical Research Council
Nakhimovsky SS., Feigl AB, Avila C, O’Sullivan G, Macgregor-Skinner E, Spranca M. (2016) [19]	Synthesis of the effectiveness of SSB taxation in middle-income countries	Primary, quantitative studies from middle-income countries (based on World Bank definitions) Reported association of taxes on or prices of SSBs with consumption and/or weight-related measures Written in English Included a published working paper and a published dissertation	January 1990–February 2013	9 (9)	Price changes and taxes	Modeling, non-experimental, quasi-experimental	Brazil, Ecuador, India, Mexico, Peru, South Africa	Adapted quality checklist for food and beverage taxes and subsidies studies from prior review (e.g., number of time points, considered all SSBs or a subset); also assessed quality of statistical methods	Independent Research and Development Grant from Abt Associates
Powell LM, Chriqui JF, Khan T, Wada R, Chaloupka FJ. (2013) [20]	Synthesis of price elasticity of demand for SSBs with calculation of summary measures	Used U.S. data Peer-reviewed (exception for USDA Economic Research Service studies) Provided original quantitative evidence on the association between prices/taxes/subsidies and consumption or weight outcomes Assessed demand for product categories (i.e., regular carbonated soda) rather than brands (i.e., Coke or Pepsi) Contained direct estimates for weight outcomes	January 2007–March 2012	21 (41)	Price changes and taxes	Cross-sectional, longitudinal	U.S.; Children and adults, aged 3 years and up	Not stated	Robert Wood Johnson Foundation, National Institutes of Health

^1^ The inclusion criteria listed describe the characteristics of the articles considered for inclusion in each review. ^2^ Relevant studies were those with a focus on interventions related to sugars. For articles that had a focus broader than sugars or examined studies other than interventions (e.g., on associations between consumption of sugars and weight), the number of relevant studies for this review may be smaller than the total number of articles reviewed. ^3^ SSBs: sugar-sweetened beverages. ^4^ RCTs: randomized controlled trials.

**Table 2:** Overview of evidence on interventions influencing price (*n* = 6 reviews).

Authors	Number of Relevant Studies Reviewed ^1^ (Locations)	Examples of Interventions Included	Study Population	Main Conclusions Regarding Effectiveness	Key Findings Related to Compensatory or Substitution Behaviors	Key Findings Related to Differential Effects by Setting/Population	Limitations, Caveats and Gaps
Backholer et al. (2016)	11 (Australia, Ireland, New Zealand, U.K., U.S.)	Taxes or price increases on SSBs ^2^	Children and adults, all ages, studies including markers of socioeconomic position	- SSB taxation is associated with improvements in population weight outcomes across socio-economic position groups or of a greater magnitude for lower compared with higher socio-economic position households	- Not addressed in detail	- Lower-income households pay a greater proportion of their income in tax, but burden across all households is small	- Variation in price elasticity estimates across studies but consistent findings of benefits related to reducing SSB consumption
Bes-Rastrollo et al. (2016)	24 (Australia, India, Ireland, New York City, South Africa, U.K., U.S)	Taxes or price increases on SSBs	Children and adults, all ages	- Studies based on simulations suggest an inverse relationship between taxes and weight gain/obesity, though magnitude of effects was small (though potential for benefit at the population level) - Observational studies (cross-sectional and cohorts) found no association between SSB taxes and weight gain	- Consumers may substitute foods with high fat or sodium content for SSBs - Could promote potential substitutions for other sugary beverages	- Some evidence that health implications may be progressive because low-income groups are more sensitive to price changes	- 18 of 24 studies were based on simulations/theoretical results - Low tax rates in real-world observational studies - Potential for price endogeneity bias
Cabrera Escobar et al. (2013)	9 (Brazil, France, Mexico, U.S.)	Taxes or price increases on SSBs	Children and adults, all ages	- Higher SSB prices associated with lower SSB demand - Studies reporting weight outcomes too heterogeneous to be pooled but those from USA suggest that higher prices associated with decrease in BMI	- Higher SSB prices associated with increased demand for alternative beverages (e.g., whole milk, fruit juices) and reduced demand for diet drinks	- Evidence from Mexico and Brazil consistent with those from higher-income countries - Evidence from Brazil suggests that lower-income individuals are more price sensitive	- Heterogeneity of study methods limit ability to synthesize and warrants caution in interpretation - Some conclusions based on small number of studies/scarce data
Levy et al. (2011)	3 (U.S.)	Taxes on SSBs, excluding SSBs from sales tax exclusion	Children, grades 1–12 (focus on youth but also discussion of adults in relation to taxation)	- Demand studies generally found that price affects soda consumption - No study found a substantial effect of soda prices on BMI	- Some evidence of offsetting effects in studies of adults	- Evidence from one study of young children found limited effects of taxes on soda consumption or weight but suggests stronger effects on those who have a higher income or higher BMI	- Heterogeneity of methods limited ability to synthesize - Studies have considered small variations in tax rates applied to a limited set of SSBs
Nakhimovsky et al. (2016)	9 (Brazil, Ecuador, India, Mexico, Peru, South Africa)	Taxes or price increase on SSBs, price reductions on SSBs	Adults, aged 19–49 years	- Higher SSB prices were associated with lower SSB consumption (decreases ranging from 5 to 39 kJ per person per day given 10% price increase) - Some indication that groups with lower socioeconomic status are more responsive to price changes in middle-income countries - Estimates consistent despite variations in baseline prevalences of obesity and per person per day consumption of SSBs	- Milk suggested to be a likely substitute; evidence on juice and alcoholic beverages unclear - Foods prepared away from home, snacks, and candy are likely complements to SSBs	- Lower-income households were more responsive to SSB taxes	- Some studies did not control for potential confounders - Small number of studies from middle-income countries - Differences in outcome measures - Variation in classification of SSBs across studies
Powell et al. (2013)	14 (U.S.)	Taxes on or price changes to SSBs	Children and adults, aged 3 years and up	- Higher SSB prices associated with lower SSB demand - Mean SSB price elasticity estimate of 1.21; a tax raising price of SSBs by 20% would reduce consumption by 24% - Evidence of impact of price changes on weight outcomes mixed	- Not addressed in detail	- Not addressed in detail due to limitations in data - One study suggested differential effects by income and race/ethnicity	- Cross-sectional evidence may overstate associations (observed for fast food prices), though significant effects observed in longitudinal studies

^1^ Relevant studies were those with a focus on interventions related to sugars. For articles that had a focus broader than sugars or that examined studies other than interventions (e.g., on associations between consumption of sugar and weight), the number of relevant studies for this review may be smaller than the total number of articles reviewed. ^2^ SSBs: sugar-sweetened beverages.

**Table 3:** Overview of evidence on interventions influencing changes to the food environment (*n* = 7 reviews).

Authors	Number of Relevant Studies Reviewed ^1^ (Location)	Examples of Interventions Included	Study Population	Main Conclusions Regarding Effectiveness	Key Findings Related to Offsetting or Compensatory Behaviors	Key Findings Related to Differential Effects by Setting/Population	Limitations, Caveats and Gaps
Althuis et al. (2013)	10 (countries not listed; follow-ups ranged 6–24 months)	Home delivery of non-caloric beverages, motivational calls/visits/advice	Children and adults, aged 6–79 years	- Delivery of beverages resulted in reduced consumption of SSBs ^2^ in the intervention group	- No discussion of compensatory behaviors	- Little discussion of differential effects	- Heterogeneity in weight-related outcome measures - Majority of articles reviewed were from European or South American countries - Little discussion of community/local context
Avery et al. (2015)	3 (Germany, Netherlands, U.S.; follow-ups ranged 11–24 months)	Home and school delivery of low-calorie beverages, changes to school food environment combined with education to increase water consumption (e.g., providing reusable water bottles to students, installing water fountains)	Children aged 2–18 years (mean age, 10 years)	- Modifying the school food environment can result in reduced SSB consumption among children in a cost-effective way - Not all studies found a reduction in BMI associated with reduced SSB consumption	- Switching from SSBs to water may be too difficult for children, suggest a move to diet/lower calorie options instead	- Reductions in SSB consumption were found across income groups, suggesting that food environment interventions could reduce health inequities among children - May be most effective for “at risk” populations (e.g., children who are already overweight)	- No studies conducted on pre-school aged children - Only one study had a follow-up and at two years, found that reductions in BMI were no longer significant - High participant attrition in home delivery study - Mostly self-reported dietary outcomes
Gibson (2008)	3 (Brazil, U.K., U.S.; 1 study included a 12–24 month follow-up)	Home delivery of non-caloric beverages, restricting availability of SSBs in schools	Children, aged 9–18 years	- Home delivery of low-calorie beverages resulted in decreased SSB consumption and weight loss in intervention group - Changes in school availability of SSBs resulted in decreased SSB consumption, but no changes in weight	- Compensatory behaviors may be impacting weight outcomes, but few studies adequately measured consumption of other foods and beverages	- Interventions may have a greater impact on “at risk” children who are already affected by overweight or obesity	- Little consideration of physical activity levels, baseline diet and BMI, ethnicity, and potential misreporting - Heterogeneity in SSB definitions and serving sizes
Levy et al. (2011)	26 (Brazil, Belgium, Canada, Holland, UK, U.S.; follow-up periods unclear)	Policies restricting SSB availability in schools (e.g., no “pouring rights” policies, restriction of SSBs in cafeterias/vending machines, reduction of SSB serving sizes)	Children, grades 1–12	- School policies that directly target the availability of SSBs in schools (e.g., vending machines, snack bars, a la carte) are associated with reduced consumption of SSBs; stricter policies appear to be more effective - It is unclear whether broad school nutrition policies (e.g., discouraging unhealthy foods in general) are effective at reducing consumption of SSBs	- Several studies did not find increased SSB consumption at home as a result of decreased consumption at school, suggesting that compensatory behaviors may not be an issue	- SSB consumption increases as children get older - Greater SSB reductions found among girls and non-Hispanic black students - Students in the National School Lunch Program have lower SSB consumption compared to other students	- Many school policies were limited in scope - No follow-up periods looked at the effect of policies beyond two years - Little consideration of school/community context - Wide variation in dose of intervention - Mostly self-reported dietary outcomes
Malik et al. (2006)	1 (U.S.)	Home delivery of non-caloric beverages and telephone contact	Children, aged 7–18 years	- Home delivery of low-calorie beverages resulted in decreased SSB consumption and weight reduction in intervention group	- May be confounding due to inadequate measurement of other diet factors	- Greater impact of intervention may be seen among children with a higher BMI at baseline	- Interpretation complicated by small sample sizes, short duration of follow-up, lack of repeated measures in dietary exposures and outcomes
Malik et al. (2013)	3 (Netherlands, U.S.; follow-ups ranged 25 weeks–18 months)	Home and school delivery of non-caloric beverages, motivational calls/visits/advice	Children, aged 8–16 years	- Home delivery of SSBs resulted in significant weight reduction in intervention group - Interventions need to be sustained to ensure a lasting impact	- Some evidence of compensatory behaviors, specifically fruit juices	- Children who were affected by overweight or obesity at baseline showed greater reductions in BMI	- Heterogeneity of studies limits the strength of summary estimates - Weight gain in children varies based on age and maturation - Little detail on serving sizes of SSBs
Mazarello et al. (2015)	12 (Australia, Asia, Belgium, Spain, U.K., U.S.; follow-ups ranged 6 months–4 years)	Reduced availability of SSBs at home combined with education	Children, aged 2.3–7 years	- Six of 12 intervention studies targeting either parents or multiple levels (child, parent, school setting) found a reduction in SSB consumption (unable to tease apart effect of food environment vs. education interventions)	- In cross-sectional analyses, milk/water consumption was not associated with reduced SSB consumption - No other discussion of compensatory behaviors	- Parental age, education, and SES were associated with higher SSB consumption in children - No other discussion of differential effects	- No detail on broader context (e.g., within the community) - Little detail on dietary outcome measurement - Majority of research came from developed countries, may not be transferable to other settings

^1^ Relevant studies were those with a focus on interventions related to sugars. For articles that examined studies other than interventions (e.g., on associations between consumption of sugar and weight), the number of relevant studies for this review may be smaller than the total number of articles reviewed. ^2^ SSBs: sugar-sweetened beverages.

**Table 4:** Overview of evidence on health promotion and education interventions (*n* = 7 reviews).

Authors	Number of Relevant Studies Reviewed ^1^ (Location)	Examples of Interventions Included	Study Populations	Main Conclusions Regarding Effectiveness	Key Findings Related to Offsetting or Compensatory Behaviors	Key Findings Related to Differential Effects by Setting/Population	Limitations, Caveats and Gaps
Althuis et al. (2013)	Unclear, studies on educational interventions and education combined with environmental interventions (countries not listed; follow-ups ranged from 6–24 months)	Educational strategies to discourage consumption of SSBs ^2^, dietary counselling	Children and adults, aged 6–79 years	- Did not report on effectiveness of educational interventions or education combined with environmental interventions (focused on evidence mapping)	- No discussion of compensatory behaviors	- Little discussion of differential effects	- Heterogeneity in weight-related outcome measures - Majority of articles reviewed were from European or South American countries - Little discussion of community/local context
Avery et al. (2015)	8 (Brazil, England, Germany, the Netherlands; follow-ups ranged 4–36 months)	Educational strategies to discourage SSB consumption (e.g., encouraging water consumption in place of SSBs, educational sessions by trained nutritionists for students AND parents/teachers, emphasis on healthy eating, reducing SSBs and sugary snacks)	Children aged 2–18 years (mean age, 10 years)	- Educational interventions of medium intensity (between 4 and 10 1-h sessions delivered over a period ranging between 6 weeks and 12 months) can be effective at reducing SSB consumption in children - Some evidence of reduced BMI, but this is not consistent across studies	- Several studies noted increased fruit and fruit juice intake among children	- Children who were affected by overweight or obesity at baseline experienced greater reductions in BMI - Greater reductions in BMI observed in girls - Some evidence of intervention efficacy across SES ^3^ groups	- Issues with non-participation by children affected by obesity, participant attrition, and underreporting (related to self-reported dietary outcome measures) - No interventions on pre-school aged children - In many studies, SSB reduction/BMI changes were not maintained over time
Gibson (2008)	1 (U.K.; 1 year follow-up)	Educational strategies to discourage SSB consumption	Children, aged 9–18 years	- Reduced consumption of SSBs in the intervention group - No significant change in mean BMI	- Substitution effects may have been an issue, but there were inadequate data gathered on other dietary factors	- No discussion of differential effects	- Children in the intervention group still gained weight, despite reducing SSB consumption - Little consideration of physical activity levels, baseline diet and BMI, ethnicity, and potential misreporting - At 2-year follow-up, there was no difference between intervention and control groups
Levy et al. (2011)	5 (Belgium, Brazil, Canada, U.K., U.S.; 2 studies included follow-ups, ranged 1–3 years)	Educational strategies to discourage SSB consumption within schools (e.g., banners and branded water bottles)	Children, grades 1–12	- Cross-sectional studies found no relationship between SSB consumption and educational programs - RCTs ^4^ found that SSB consumption was reduced following educational interventions, inconsistent evidence for reduction in BMI	- No discussion of compensatory behaviors	- Reduction in BMI only significant among girls - Greater reductions in BMI observed among children who were already affected by overweight or obesity, but these reductions were not maintained at follow-up	- Self-reported dietary outcome measures - Lack of adequate follow-up periods - None of the educational programs targeted only SSBs - No consideration of race/ethnicity, SES or other demographics - Little consideration of environmental context (e.g., community/region)
Malik et al. (2006)	1 (U.S.; 1 year follow-up)	Educational strategies to reduce SSB consumption in a school	Children, aged 7–18 years	- A modest reduction in SSB consumption and a reduction in prevalence of overweight/obesity in the intervention group	Not stated	Not stated	- Interpretation of the published studies complicated by method-related issues, including small sample sizes, short duration of follow-up, lack of repeated measures in dietary exposures and outcomes
Malik et al. (2013)	2 (Brazil, U.K.; 1 year follow-up)	Educational strategies to discourage SSB consumption within schools	Children, aged 8–16 years	- No significant effect of educational interventions on SSB consumption - No sustained effect on weight	- Students may have been substituting fruit juices and other sugary drinks for SSBs	- Interventions may be more effective for children who are already affected by overweight/obesity	- Heterogeneity of studies limits the strength of summary estimates - Little detail on serving sizes of SSBs
Mazarello et al. (2015)	12; unclear how many studies focused on educational interventions (countries not listed; follow-ups ranged 6 months–4 years)	Educational strategies to improve diet and physical activity	Children, aged 2.3–7 years	- Six of 12 intervention studies targeting either parents or multiple levels (child, parent, school setting) found a reduction in SSB consumption (unable to tease apart effect of environment vs. education interventions)	- Milk/water consumption was not associated with reduced SSB consumption - No other discussion of compensatory behaviors	- Parental age, education, and SES were associated with higher baseline SSB consumption - No other discussion of differential effects	- No detail on broader context (e.g., within the community) - Very little detail on dietary outcome measurement - Majority of research came from developed countries, may not be transferable

^1^ Relevant studies were those with a focus on interventions related to sugars. For articles that had a focus broader than sugars or that examined studies other than interventions (e.g., on associations between consumption of sugar and weight), the number of relevant studies for this review may be smaller than the total number of articles reviewed. ^2^ SSBs: sugar-sweetened beverages. ^3^ SES: socioeconomic status. ^4^ RCTs: randomized controlled trials.

## Supplementary Materials

The following are available online at http://www.mdpi.com/2072-6643/10/8/1036/s1, File S1: Search Strategy, File S2: PRISMA 2009 Checklist.
