# Supplementary material for: Gaps in the Evidence on Population Interventions to Reduce Consumption of Sugars: A Review of Reviews
Source: Nutrients. 2018 Aug 8;10(8):1036. doi: 10.3390/nu10081036 (PMC6115867; doi:10.3390/nu10081036)
Supplement: Supplementary file 1 [file nutrients-10-01036-s001.zip › nutrients-331229-supplementary/File S1 Search Strategy.docx]

Review

Gaps in the evidence on population interventions to reduce consumption of sugars: A review of reviews

Sharon I. Kirkpatrick ^1^*, Amanda Raffoul ^1^, Merryn Maynard ^2^, Kirsten M. Lee ^1^, Jackie Stapleton ^3^

Supplemental Material

Search Strategy

**Example headings and keywords: MEDLINE**

The following MeSH terms and keywords for searching titles and abstracts were selected to capture review articles published in English since January 2005 that focus on interventions to reduce sugar intake. Three main concepts are captured: sugar, interventions, and reviews.

**Search term table:**

**Sugar**

| .mp. fields^1^ | | MeSH subject headings |
| --- | --- | --- |
| sweet*  sugar*  sucrose  discretionary calorie*  energy dense  junk food  soft drink*  cake*  pastry  pastries  biscuit*  pudding*  jam  jams  marmalade*  confectioner*  chocolate* | energy drink*  sports drink*  sport drink*  yogurt  yoghurt  breakfast cereal*  juice*  snack*  candy  candies  dessert*  fizzy  soda  pop | sucrose  sweetening agents  high fructose corn syrup |

^1^In Medline: mp=title, abstract, original title, name of substance word, subject heading word, keyword heading word, protocol supplementary concept word, rare disease supplementary concept word, unique identifier. *Wildcard.

**Interventions**

| .ti. = title only** | .tw. = title, abstract | MeSH subject headings |
| --- | --- | --- |
| prevent* or program* or strategy or strategies | intervention* or policy or policies or initiative* or guideline* | child health services  health promotion  health education  primary prevention  preventive health services  school health services consumer health information health education, dental  health fairs  nutrition assessment  nutrition surveys  policy making  health policy  nutrition policy |
|  | nutrition label* or calorie label* or reformulation* or nutrition* composition or nutrient list* or nutrition claim* or health claim* or nutrient content* or nutrient information or food label* | food labeling  nutritive value |
|  | tax or taxes or taxation or fiscal* or price or pricing or prices or tariff* or subsid* or incentiv* or econometric* | food/economics  diet/economics  taxes  financing, government |
|  | advertis* or product placement or television or marketing or mass media or social media or campaign* | marketing or mass media |
|  | behaviour change or behavior change |  |

*Wildcard.

**The search for these terms is limited to the title field because these are very common words and an abstract search retrieved many articles that were not focused on interventions.

**Reviews**

| .ti. = title only | MeSH subject headings |
| --- | --- |
| review or reviews or synthesis or overview or meta analysis | review  meta analysis |

**Limits:**

- English only;
- Publication date 2005-current;
- Human studies only;
- Exclude metabolism studies (except for dietary sucrose metabolism); and,
- Exclude journals that contain either ‘chemistry’ or ‘biochemistry’ in the journal title to eliminate articles that do not pertain to interventions and strategies in human populations.

MEDLINE (OVID): performed by Jackie Stapleton, November 19, 2015 and May 5, 2017

| 1 | (discretionary calorie* or energy dense or soft drink* or cake* or pastry or pastries or biscuit* or pudding* or jam or jams or marmalade* or confectioner* or chocolate* or energy drink* or sports drink* or sport drink* or yogurt or yoghurt or breakfast cereal* or juice* or snack* or candy or candies or dessert* or fizzy or soda or pop or junk food).mp. |
| --- | --- |
| 2 | carbonated beverages/ or energy drinks/ or exp candy/ or yogurt/ or snacks/ |
| 3 | review/ or meta analysis/ or (review or reviews or synthesis or overview or meta analysis).ti. |
| 4 | (sweet* or sugar* or sucrose).mp. or exp sucrose/ or exp sweetening agents/ or high fructose corn syrup/ |
| 5 | 1 or 2 or 4 |
| 6 | exp child health services/ or exp health promotion/ or exp health education/ or primary prevention/ or secondary prevention/ or preventive health services/ or exp school health services/ or exp consumer health information/ or health education, dental/ or health fairs/ or community health services/ or nutrition assessment/ or exp nutrition surveys/ or policy making/ or health policy/ or nutrition policy/ or health behavior/ or exp obesity/pc |
| 7 | (prevent* or program* or strategy or strategies).ti. or (intervention* or policy or policies or initiative* or guideline* or health behavior or health behaviour).tw. |
| 8 | (nutrition label* or calorie label* or reformulation* or nutrition* composition or nutrient list* or nutrition claim* or health claim* or nutrient content* or nutrient information or food label*).tw. or food labeling/ or nutritive value/ |
| 9 | (tax or taxes or taxation or fiscal* or price or pricing or prices or tariff* or subsid* or incentiv* or econometric*).tw. or food/ec or diet/ec or taxes/ or financing, government/ |
| 10 | (advertis* or product placement or television or marketing or mass media or social media or campaign* or cafeteria*).tw. or exp marketing/ or mass media/ |
| 11 | 6 or 7 or 8 or 9 or 10 |
| 12 | 3 and 5 and 11 |
| 13 | limit 12 to english language |
| 14 | exp Eukaryota/ not humans/ |
| 15 | (chemistry or biochemistry).jw. |
| 16 | exp metabolism/ not dietary sucrose/me |
| 17 | 13 not (14 or 15 or 16) |
| 18 | limit 17 to yr="2005 -Current" |

EMBASE (OVID): performed by Jackie Stapleton, November 20, 2015 and May 15, 2017

| 19 | (soft drink* or cake* or pastry or pastries or biscuit* or pudding* or jam or jams or marmalade* or confectioner* or chocolate* or energy drink* or sports drink* or sport drink* or yogurt or yoghurt or breakfast cereal* or juice* or snack* or candy or candies or dessert* or fizzy or soda or pop or junk food*).mp. |
| --- | --- |
| 20 | exp carbonated beverage/ or soft drink/ or fruit juice/ or energy drink/ or sports drink/ or fast food/ or yogurt/ |
| 21 | review/ or meta analysis/ or (review or reviews or synthesis or overview or meta analysis).ti. or systematic review.mp. |
| 22 | (sweet* or sugar* or sucrose).mp. or sugar/ or sucrose/ or exp sweetening agent/ or sugar intake/ |
| 23 | 19 or 20 or 22 |
| 24 | health promotion/ or exp health education/ or primary prevention/ or prevention/ or preventive health service/ or consumer health information/ or exp community care/ or community medicine/ or nutritional assessment/ or health survey/ or health care policy/ or standards/ or government regulation/ or health behavior/ or harm reduction/ or exp obesity/pc |
| 25 | (prevent* or program* or strategy or strategies).ti. or (intervention* or policy or policies or initiative* or guideline* or health behavior or health behaviour).tw. |
| 26 | (nutrition label* or calorie label* or reformulation* or nutrition* composition or nutrient list* or nutrition claim* or health claim* or nutrient content* or nutrient information or food label*).tw. or food packaging/ or nutritional value/ |
| 27 | (tax or taxes or taxation or price or pricing or prices or tariff*).tw. or tax/ or cost benefit analysis/ or cost control/ or cost effectiveness analysis/ or food subsidies/ |
| 28 | (advertis* or product placement or television or marketing or mass media or social media or campaign* or cafeteria*).mp. or marketing/ or advertizing/ or exp mass communication/ |
| 29 | 24 or 25 or 26 or 27 or 28 |
| 30 | 21 and 23 and 29 |
| 31 | (exp animal/ or nonhuman/) not exp human/ |
| 32 | (chemistry or biochemistry or biotechnology).jw. |
| 33 | abstract.pt. |
| 34 | 30 not (31 or 32 or 33) |
| 35 | limit 34 to yr="2005 -Current" |
| 36 | limit 35 to english language |

CINAHL (EBSCOHost): performed by Jackie Stapleton, November 20, 2015 and May 5, 2017

| S8 | S1 AND S6 AND S7  , **Limiters** - Published Date: 20050101-20170631; English Language |
| --- | --- |
| S7 | ( (MH "Literature Review+") OR (MH "Systematic Review") OR (MH "Meta Analysis") ) OR PT review OR TI ( review or reviews or synthesis or overview or "meta analysis" ) |
| S6 | S2 OR S3 OR S4 OR S5 |
| S5 | ( (MH "Telecommunications+") OR (MH "Social Media") OR (MH "Marketing+") ) OR TI ( advertis* or "product placement" or television or marketing or "mass media" or "social media" or campaign* or cafeteria* ) OR AB ( advertis* or "product placement" or television or marketing or "mass media" or "social media" or campaign* or cafeteria* ) |
| S4 | ( (MH "Sucrose/EC") OR (MH "Dietary Sucrose/EC") OR (MH "Taxes") OR (MH "Financing, Government") ) OR TI ( tax or taxes or taxation or fiscal* or price or pricing or prices or tariff* or subsid* or incentiv* or econometric* ) OR AB ( tax or taxes or taxation or fiscal* or price or pricing or prices or tariff* or subsid* or incentiv* or econometric* ) |
| S3 | ( (MH "Food Labeling") OR (MH "Nutritive Value") ) OR TI ( "nutrition label*" or "calorie label*" or reformulation* or "nutrition* composition" or "nutrient list*" or "nutrition claim*" or "health claim*" or "nutrient content*" or "nutrient information" or "food label*" ) OR AB ( "nutrition label*" or "calorie label*" or reformulation* or "nutrition* composition" or "nutrient list*" or "nutrition claim*" or "health claim*" or "nutrient content*" or "nutrient information" or "food label*" ) |
| S2 | TI ( intervention* or policy or policies or initiative* or guideline* OR prevent* or program* or strategy or strategies ) OR AB ( intervention* or policy or policies or initiative* or guideline* OR prevent* or program* or strategy or strategies ) OR ( (MH "Health Promotion") OR (MH "Preventive Health Care") OR (MH "School Health Services+") OR (MH "Student Health Services+") OR (MH "Education") OR (MH "Nutritional Assessment") OR (MH "Nutrition Policy+") OR (MH "Policy Making") OR (MH "Public Policy+") OR (MH "Community Health Services+") ) |
| S1 | TI ( "discretionary calorie*" or "energy dense" or "soft drink*" or cake* or pastry or pastries or biscuit* or pudding* or jam or jams or marmalade* or confectioner* or chocolate* or "energy drink*" or "sports drink*" or "sport drink*" or yogurt or yoghurt or "breakfast cereal*" or juice* or snack* or candy or candies or dessert* or fizzy or soda or pop or "junk food" ) OR AB ( "discretionary calorie*" or "energy dense" or "soft drink*" or cake* or pastry or pastries or biscuit* or pudding* or jam or jams or marmalade* or confectioner* or chocolate* or "energy drink*" or "sports drink*" or "sport drink*" or yogurt or yoghurt or "breakfast cereal*" or juice* or snack* or candy or candies or dessert* or fizzy or soda or pop or "junk food" ) OR TI ( sweet* or sugar* or sucrose ) OR AB ( sweet* or sugar* or sucrose ) OR ( (MH "Sucrose+") OR (MH "Sweetening Agents") OR (MH "Carbonated Beverages") OR (MH "Energy Drinks") OR (MH "Fruit Juices+") OR (MH "Snacks") OR (MH "Candy") ) |

Cochrane Library: Cochrane Database of Systematic Reviews, November 20, 2015 and May 5, 2017

#1

discretionary calorie*" or "energy dense" or soft drink* or cake* or pastry or pastries or biscuit* or pudding* or jam or jams or marmalade* or confectioner* or chocolate* or "energy drink*" or "sports drink*" or "sport drink*" or yogurt or yoghurt or "breakfast cereal*" or juice* or snack* or candy or candies or dessert* or fizzy or soda or pop or "junk food":ti,ab,kw or sweet* or sugar* or sucrose or "high fructose corn syrup":ti,ab,kw (Word variations have been searched)

#2

prevent* or program* or strategy or strategies* or intervention* or policy or policies or initiative* or guideline* or "health behavior" or "health behaviour":ti or intervention* or policy or policies or initiative* or guideline* or "health behavior" or "health behaviour":ab or "child health services" or "health promotion" or "health education" or "primary prevention" or "secondary prevention" or "preventive health services" or "school health services" or "consumer health information" or "health fairs" or "nutrition assessment" or "nutrition surveys" or "communty health services" or "policy making" or "health policy" or "nutrition policy" or "health behavior":kw or "nutrition label*" or "calorie label*" or reformulation* or "nutrition* composition" or "nutrient list*" or "nutrition claim*" or "health claim*" or "nutrient content*" or "nutrient information" or "food label*":ti,ab,kw or tax or taxes or taxation or fiscal* or price or pricing or prices or tariff* or subsid* or incentiv* or econometric* or economics or financing or advertis* or "product placement" or television or marketing or "mass media" or "social media" or campaign* or cafeteria* (Word variations have been searched)

#1 and #2
